# Supplementary figures and images for: Identification of PS1/gamma-secretase and glutamate transporter GLT-1 interaction sites
Source: J Biol Chem. 2024 Mar 16;300(4):107172. doi: 10.1016/j.jbc.2024.107172 (PMC11015137; doi:10.1016/j.jbc.2024.107172)

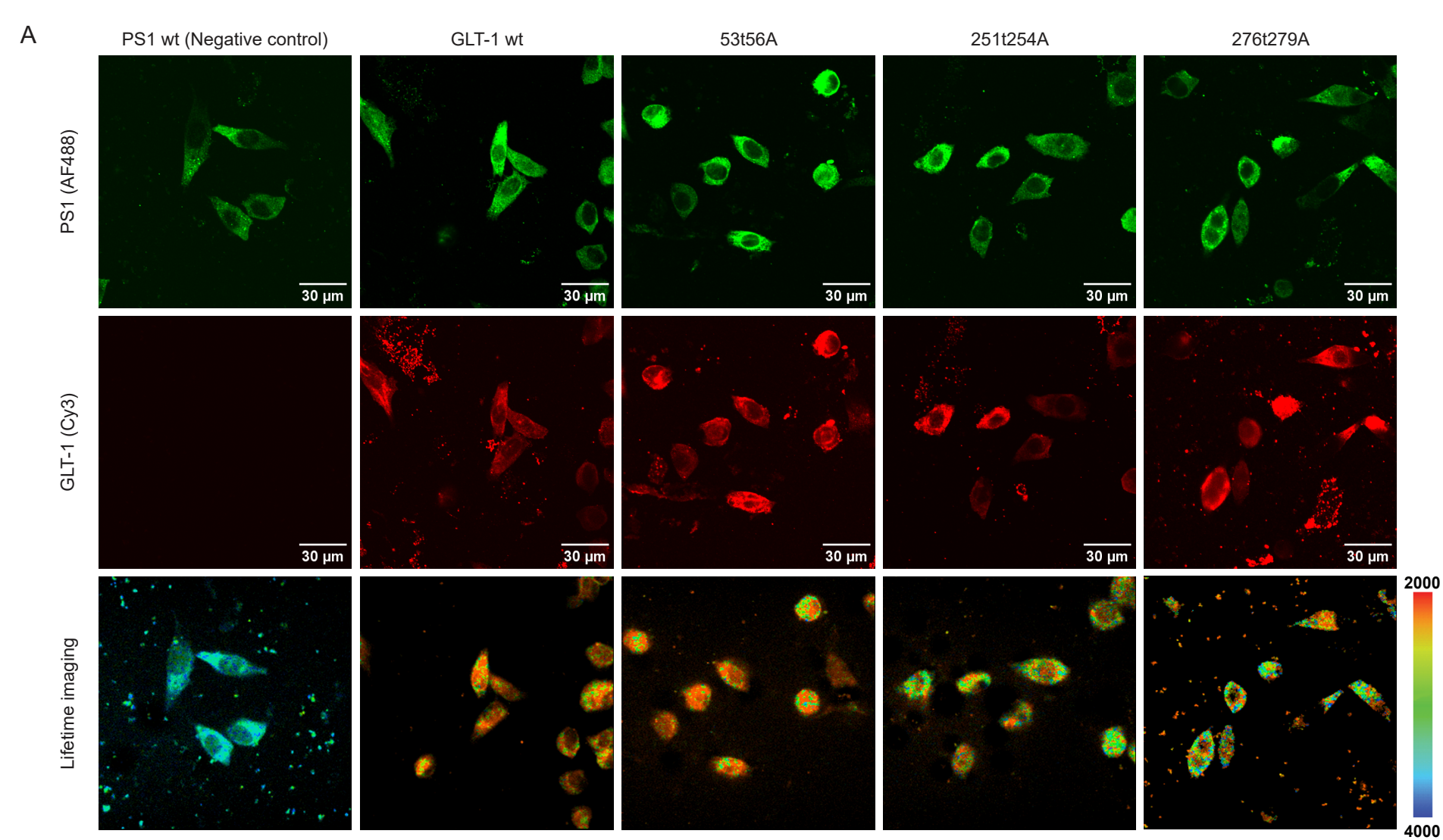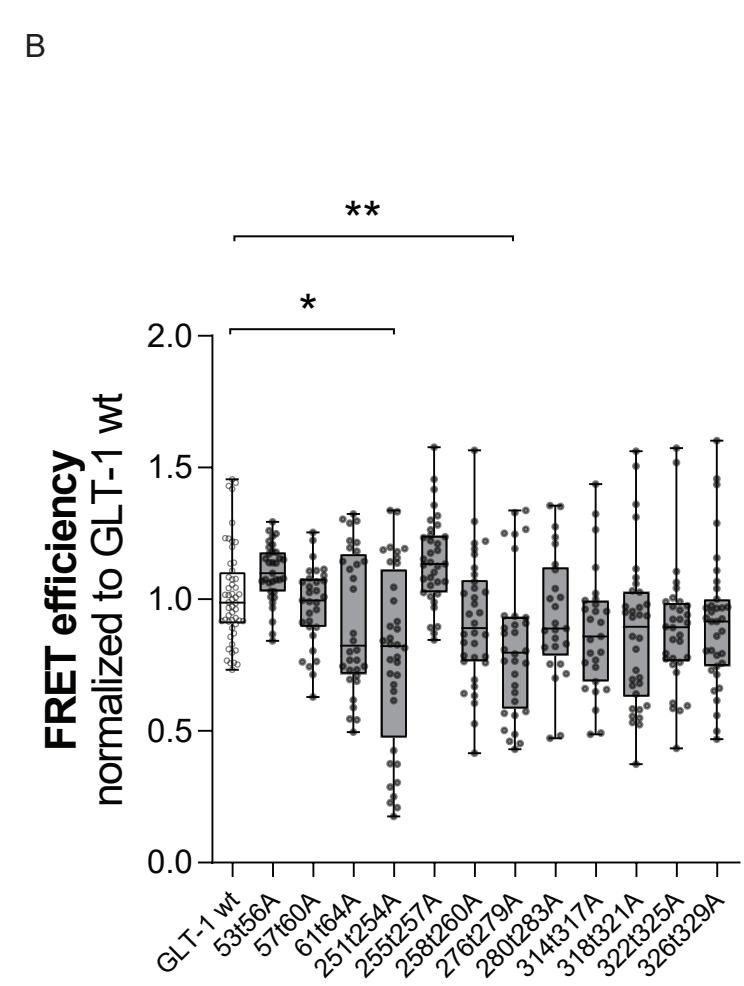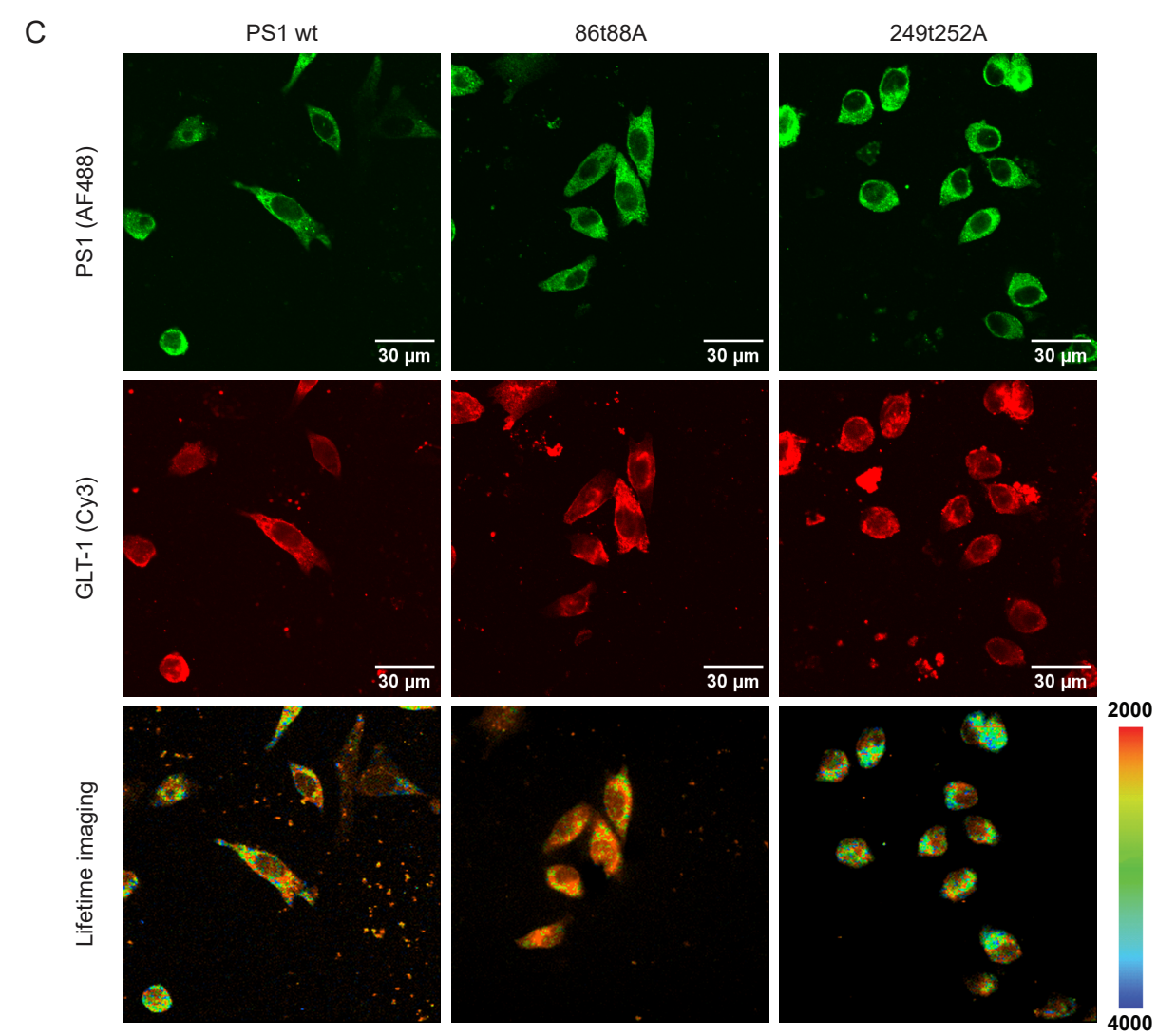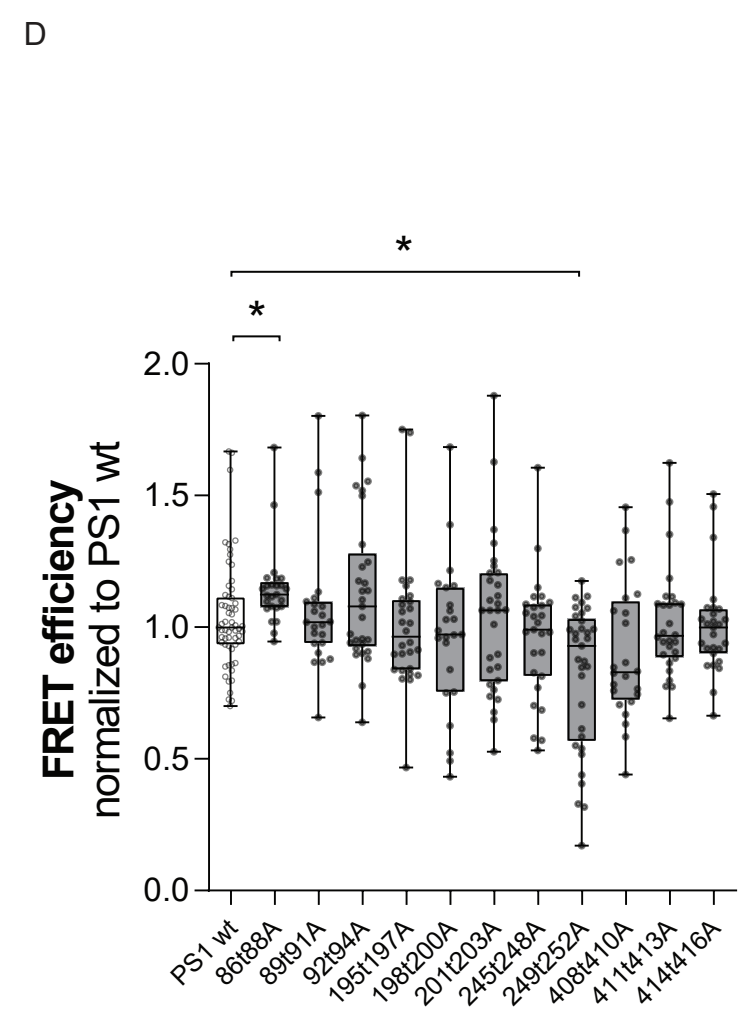

Supplement: Supporting Figure S1 [file mmc1.pdf]

A

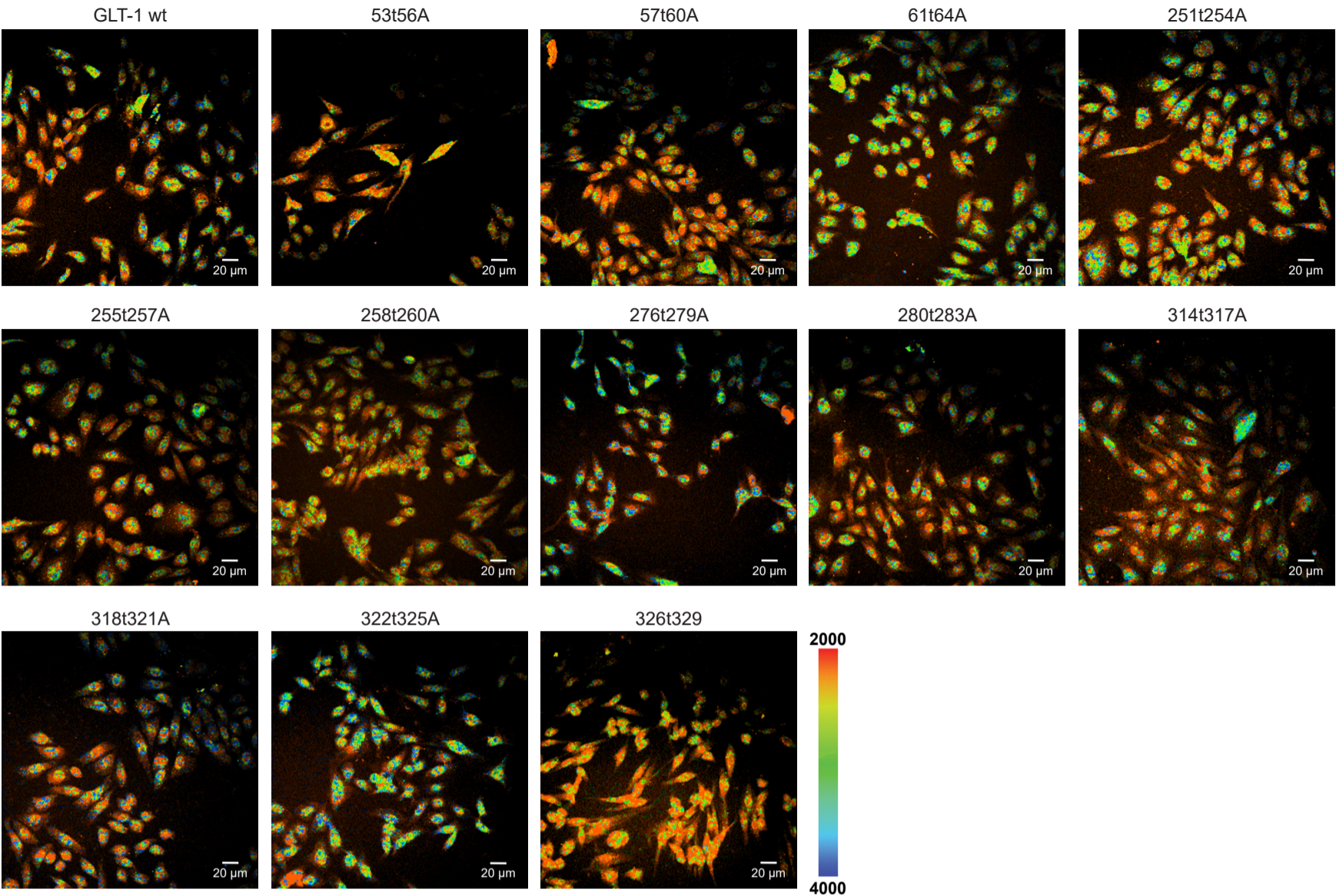

B

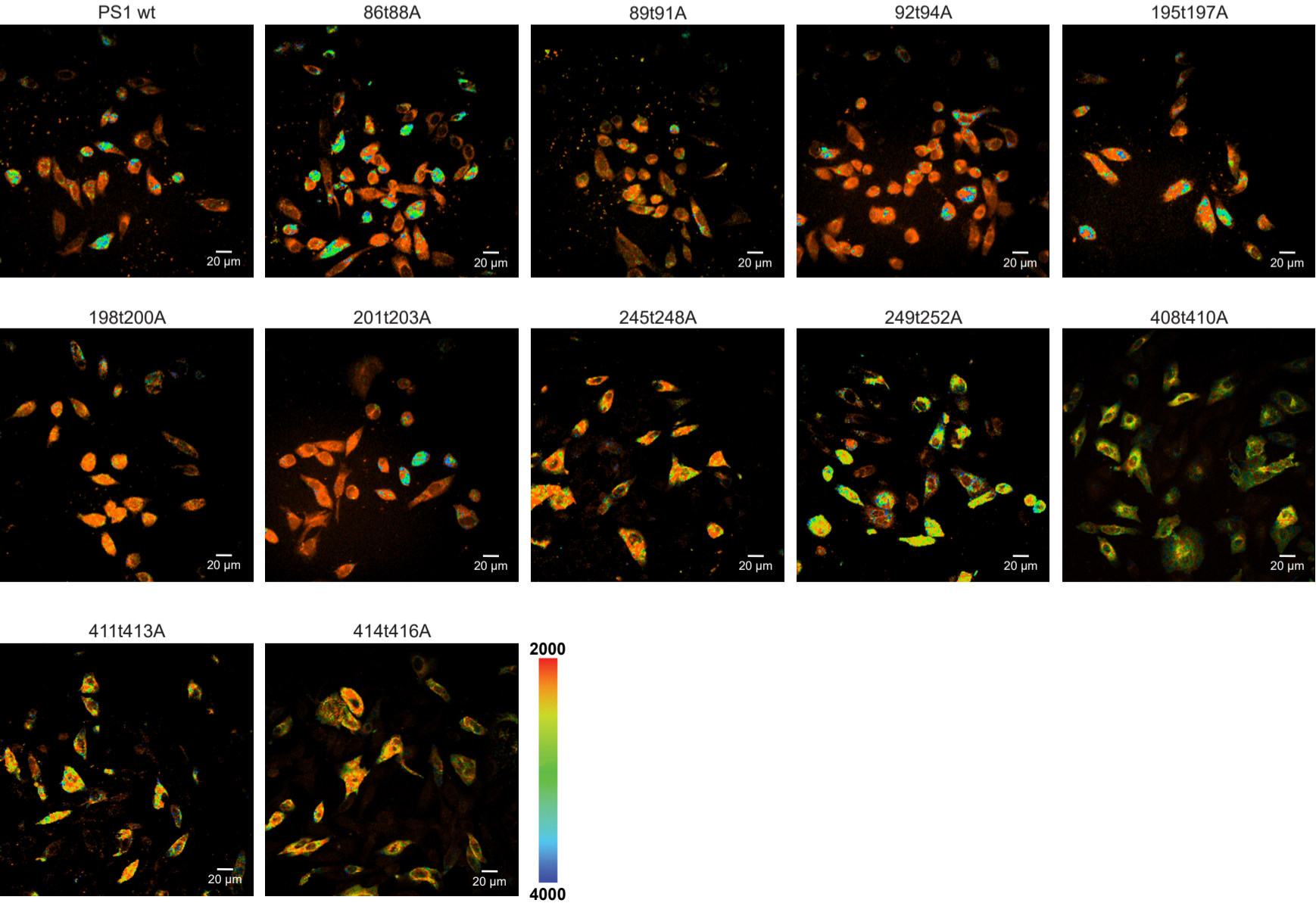

Supplement: Supporting Figure S2 [file mmc2.pdf]

A

PS1 N-term (AF488)

PS1 loop (Cy3)

Lifetime imaging

PS1 wt  
(Negative control)

PS1 wt

86t88A

249t252A

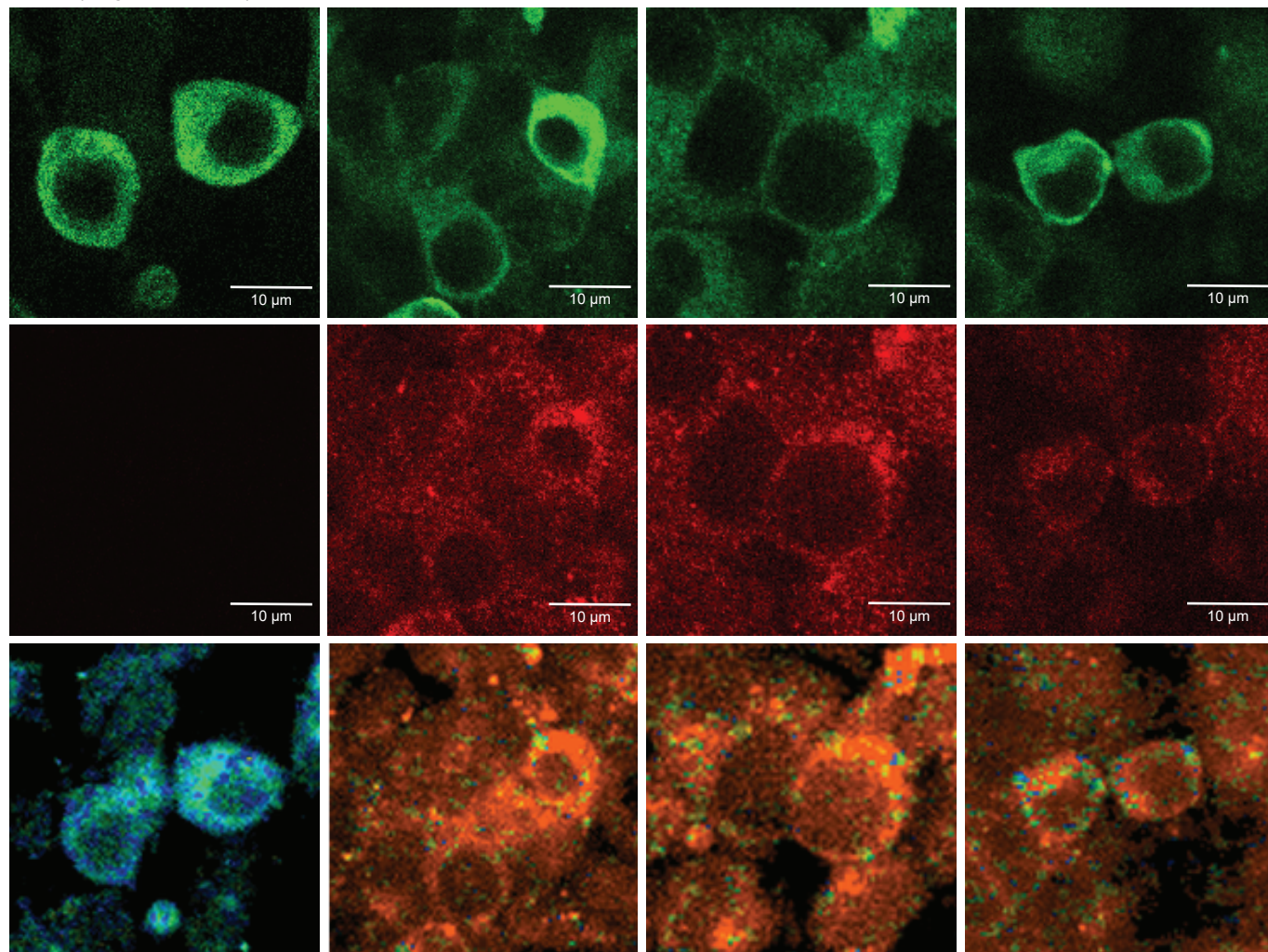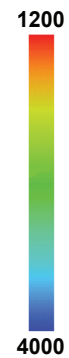

B

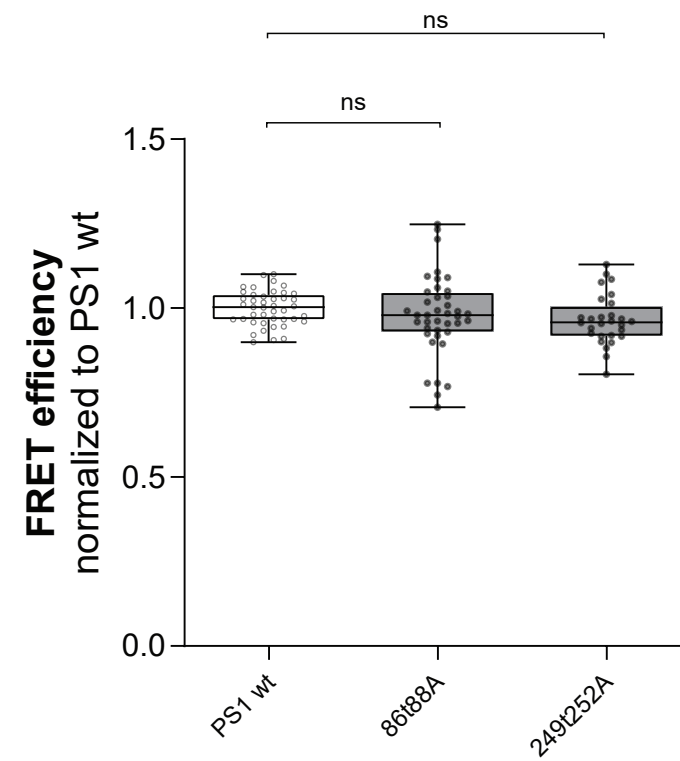

Supplement: Supporting Figure S3 [file mmc3.pdf]

A

## FAM-GLT-1 CPP

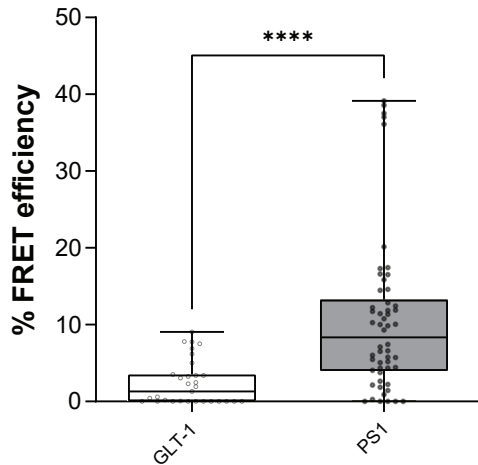

B

## FAM-PS1 CPP

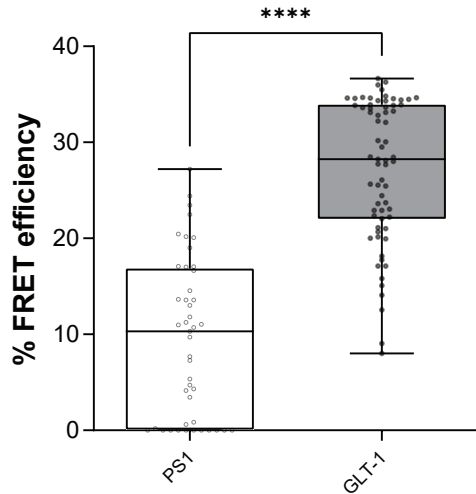

Supplement: Supporting Figure S4 [file mmc4.pdf]

Figure 1

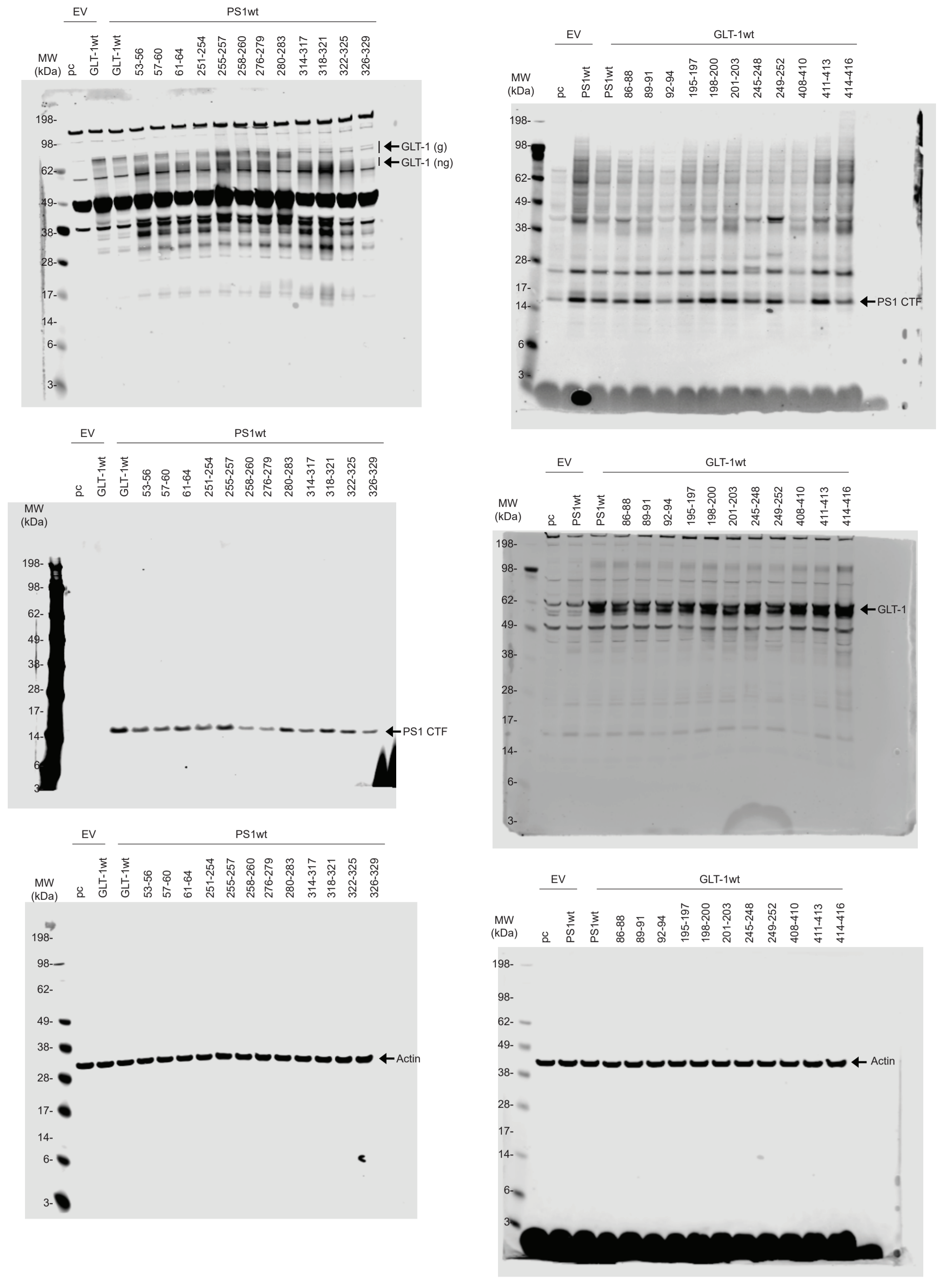

Figure 2

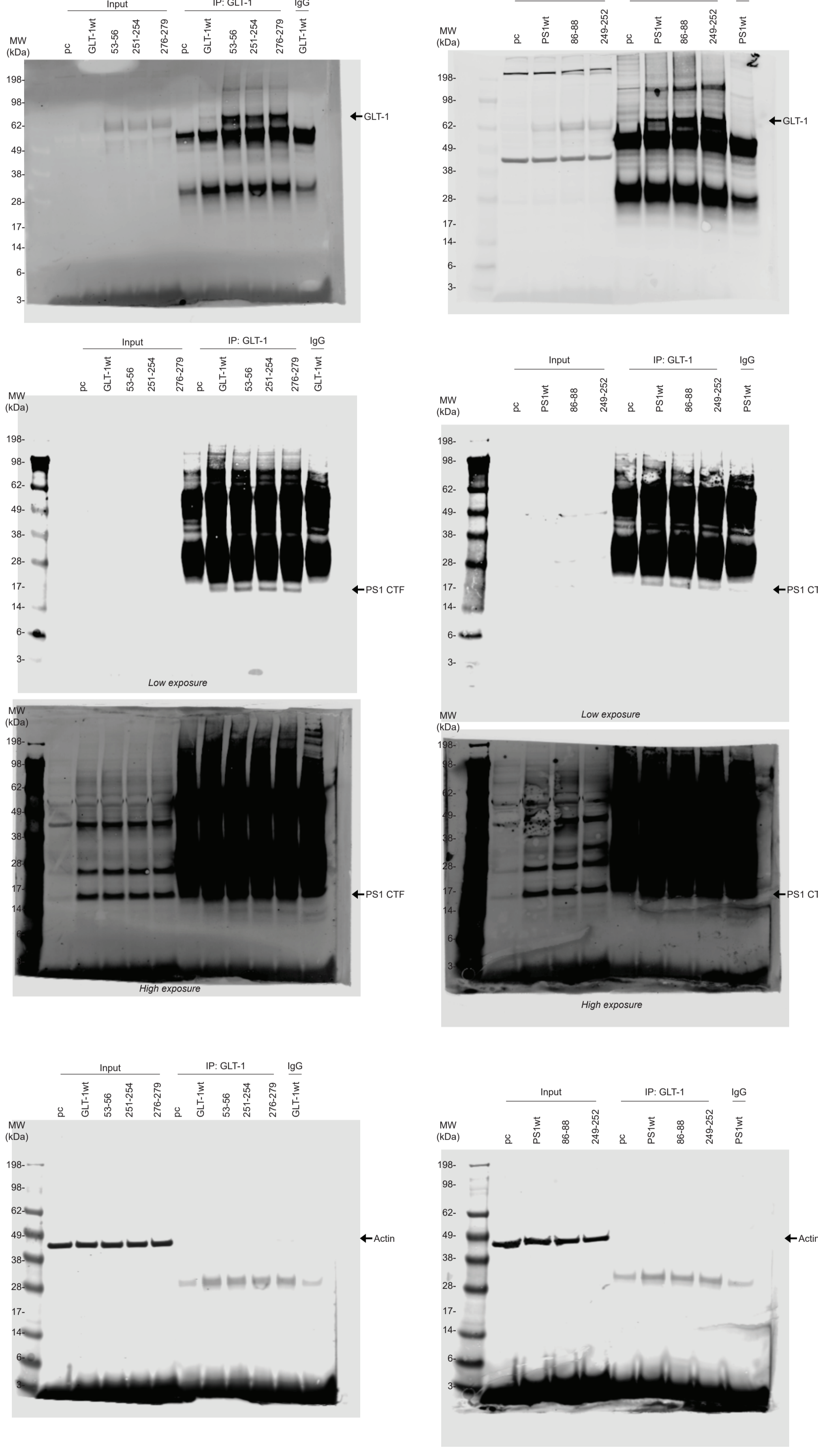

Figure 3

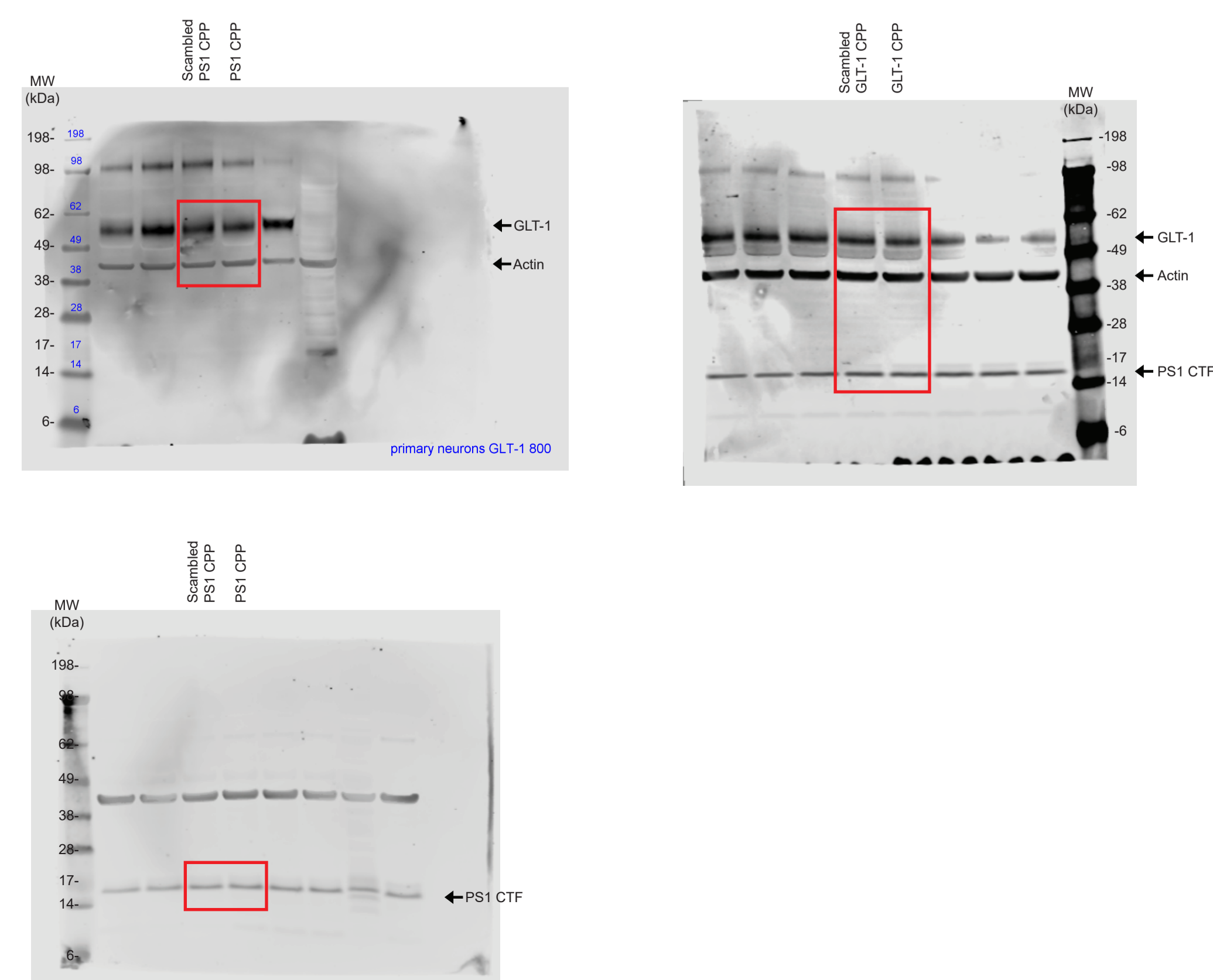

Supplement: Supporting Figure S5 [file mmc5.pdf]
