# Supplementary material for: Identification of PS1/gamma-secretase and glutamate transporter GLT-1 interaction sites
Source: J Biol Chem. 2024 Mar 16;300(4):107172. doi: 10.1016/j.jbc.2024.107172 (PMC11015137; doi:10.1016/j.jbc.2024.107172)
Supplement: Supporting Information [file mmc6.docx]

**Figure S1. PS1/GLT-1 interaction in CHO cells.** A) Confocal images show GLT-1 wt and GLT-1 alanine mutants’ immunoreactivity in CHO cells. The cells were immunostained with anti-PS1 and anti-GLT-1 antibodies, followed by AF488 and Cy3-labelled secondary antibodies, respectively. Color-coded FLIM images show AF488 PS1 donor fluorophore lifetimes, representing different proximity between PS1 and GLT-1 fluorophore. Colorimetric scale shows fluorescence lifetime in picoseconds. Longer AF488 lifetimes (blue-green pixels) represent diminished proximity, i.e., reduced interaction between PS1 and GLT-1. B) FLIM analysis of GLT-1/PS1 proximity (%E_FRET_) in CHO cells. Average %E_FRET_ in cells expressing wt GLT-1 (n=4 independent experiments, ~15 cells per ROI), and GLT-1 alanine mutants (n=4, 10~cells per ROI). Box and whiskers plot with min and max bars; Kruskal-Wallis ANOVAs with Dunn’s multiple comparison test. C) Confocal images show PS1 wt and PS1 alanine mutants’ immunoreactivity in CHO cells. The cells were immunostained with anti-PS1 and anti-GLT-1 antibodies, followed by AF488 and Cy3-labelled secondary antibodies, respectively. Color-coded FLIM images show AF488 PS1 donor fluorophore lifetimes, representing different proximity between fluorophores labeling PS1 and GLT-1. Colorimetric scale shows fluorescence lifetime in picoseconds. D) Average %E_FRET_ in cells expressing PS1wt as a control (n=4, 15~ cells per ROI), and PS1 alanine mutants (n=4 independent experiments, 10~cells per ROI). The results are expressed as FRET efficiency normalized to the corresponding wild-type protein. Box and whiskers plot with min and max bars, Kruskal-Wallis ANOVAs with Dunn’s multiple comparison test.

**Figure S2. FLIM analysis of the PS1/GLT-1 interaction.** The cells were immunostained with anti-PS1 and anti-GLT-1 antibodies, followed by AF488 and Cy3-labelled secondary antibodies, respectively. Color-coded FLIM images show AF488 PS1 donor fluorophore lifetimes, representing different proximity between PS1 and GLT-1 fluorophore. Colorimetric scale shows fluorescence lifetime in picoseconds. Longer AF488 lifetimes (blue-green pixels) represent diminished proximity, i.e., reduced interaction between PS1 and GLT-1. Pseudo-colored FLIM images show interaction between PS1 wt and GLT-1 wt/GLT-1 alanine mutants (A), and between GLT-1 wt and PS1 wt/PS1 alanine mutants (B).

**Figure S3. FLIM analysis of the PS1 conformation.** (A) HEK PS DKO were transfected with wtPS1 or PS1 alanine mutants, and immunostained with anti-PS1 N-terminus (NT) and anti-PS1 loop antibodies, followed by AF488 and Cy3-labelled secondary antibodies, respectively. PS1 loop antibody was omitted to determine baseline AF488 lifetime in FLIM negative control. Color-coded FLIM images show AF488 PS1 NT donor fluorophore lifetimes. Colorimetric scale shows fluorescence lifetime in picoseconds. representing different proximity between PS1 NT and PS1 loop fluorophore. Shortening of the AF488 lifetime in double-stained cells (orange-red pixels) indicates FRET, i.e., close proximity between PS1 NT and PS1 loop. (B) Average %E_FRET_ in cells expressing PS1wt as a control, and PS1 alanine mutants indicates relative proximity between PS1 NT and loop region (n=3 independent experiments, ~20 cells per ROI per experiment). The results are expressed as FRET efficiency normalized to PS1 wt. Box and whiskers plot with min and max bars, Kruskal-Wallis ANOVAs with Dunn’s multiple comparison test. ns: non-significant.

**Figure S4. FLIM analysis of the CPP binding to its target protein.** (A) GLT-1 CPP binding to PS1 protein in neurons was determined by FLIM. The neurons were treated for two hours with 5 μM FAM-GLT-1 CPP, fixed with PFA, and immunostained with anti-GLT-1 or anti-PS1 antibodies, followed by Cy3 conjugated secondary antibodies. The change in FRET efficiency was used to estimate relative change in the proximity between FAM tag on GLT-1 CPP and Cy3 labeled GLT-1 or Cy3 labeled PS1 proteins. The graph shows % FRET efficiencies of FAM GLT-1 CPP binding to GLT-1 or PS1 proteins. Box and whiskers plot with min and max bars. T-test followed by Mann-Whitney post hoc test is used to determine CPP’s binding preference; ****p <0.0001. B) PS1 CPP binding to GLT-1 protein in neurons was determined by FLIM. The neurons were treated for two hours with 5 μM FAM-PS1 CPP and immunostained with anti-PS1 or anti-GLT-1 antibodies, followed by Cy3 conjugated secondary antibodies. The change in FRET efficiency was used to estimate relative change in the proximity between FAM tag on PS1 CPP and Cy3-labeled PS1 or Cy3-labeled GLT-1 proteins. The graph shows % FRET efficiencies of FAM PS1 CPP binding to PS1 or GLT-1 proteins. Box and whiskers plot with min and max bars. T-test followed by Mann-Whitney post hoc test; ****p <0.0001.

**Figure S5. Images of the entire blots that were used to generate Figures 1, 2, and 3.**

MW: molecular weight in kDa
